# Clarification of Bio-Degumming Enzymes Based on a Visual Analysis of the Hemp Roving Structure

**DOI:** 10.3390/polym16243592

**Published:** 2024-12-22

**Authors:** Tianyi Yu, Pandeng Li, Tong Shu, Tingting Liu, Chunhua Fu, Longjiang Yu

**Affiliations:** 1 Department of Biotechnology, Institute of Resource Biology and Biotechnology, College of Life Science and Technology, Huazhong University of Science and Technology, Wuhan 430074, China; yu_tianyi@hust.edu.cn (T.Y.); lipandeng@hust.edu.cn (P.L.); shutong0618@126.com (T.S.); d201780454@hust.edu.cn (T.L.); yulongjiang@hust.edu.cn (L.Y.); 2 Key Laboratory of Molecular Biophysics, Ministry of Education, Huazhong University of Science and Technology, Wuhan 430074, China

**Keywords:** hemp fiber, bio-degumming, hemicellulose, pectin, degumming enzymes

## Abstract

Hemp fibers, recognized for their breathability, specific strength, and ultraviolet resistance, are widely utilized in textile manufacturing and composite materials. Bio-degumming is a promising alternative technology to traditional chemical degumming that can be used to produce hemp fibers due to its eco-friendly nature. However, its lower efficiency has hindered its widespread adoption. The unclear and complex structure of the gums leads to a poor understanding on the enzyme types required for bio-degumming, thereby restricting improvements in its efficiency. In this study, the morphological characteristics, polysaccharide composition, and branched structure of hemp stem, roving fibers, and refined fibers were investigated using scanning electron microscopy and laser scanning confocal microscopy in combination with immunofluorescence techniques, with a view to identify the enzymes necessary for the efficient bio-degumming of hemp. The results revealed that the gums were primarily located in the middle lamella, phloem parenchyma, and certain xylem tissues. These tissues showed chunk-like, fence-like, and plate-like shapes, respectively, and tightly wrapped around the fiber bundles. In these tissues, pectin comprised low-esterified homogalacturonan, along with rhamnogalacturonan carrying galactan and arabinan branches. Xylan exhibited acetyl, arabinose, and glucuronic acid branches, while mannan displayed acetyl and galactose branches. Partial xylan and mannan were masked by pectin, and the branching structures impeded their enzymatic removal. As a consequence, the necessary enzymes and their synergistic effects for effective hemp roving degumming were elucidated. Pectin degradation was facilitated by pectate lyase and rhamnogalacturonan-degrading enzymes. Xylan and mannan were effectively removed by endo-xylanase and endo-mannanase, a process necessitating the synergistic action of branched-chain-degrading enzymes, including the esterase, α-L-arabinofuranosidase, α-galactosidase, and α-glucuronidase. This study provided practical strategies to enhance the efficiency of hemp bio-degumming.

## 1. Introduction

In the context of global sustainable development, bast fibers are attracting interest as a crucial renewable resource [1]. Hemp, acknowledged as a member of the bast fiber family, is cultivated widely around the world [2,3]. The remarkable characteristics of hemp fibers, including its superior breathability, moisture absorption, antibacterial properties, and ultraviolet resistance, have facilitated its widespread application in textile manufacturing and industrial composites [4,5]. Traditionally, harvested hemp underwent a retting process and was then machined into roving in preparation for the production of fine yarns [6]. However, the fibers in the roving were tightly bound by gums, which consisted of non-cellulosic components such as hemicellulose (10–16%), pectin (4–5%), lignin (8–15%), wax (1–4%), and water-soluble substances (2–6%) [7], limiting the further utilizations in high-value textiles. Currently, alkali degumming is the predominant technology that is used in hemp fiber production due to its high degumming efficiency; however, it involves the significant consumption of alkali and water, causing pollution to the aquatic environment [8,9]. In contrast, bio-degumming utilizes enzymes or microbes capable of producing enzymes as an alternative to chemical reagents, thereby promoting energy efficiency and environmental sustainability [10].

Hemp bio-degumming methods have been optimized through various approaches. Various studies have included the screening of degumming strains [11], the development of anaerobic microbial community degumming systems [12], combined enzyme degumming strategies [13], the immobilization of enzymes for degumming [14], and the integration of enzymes with chemical auxiliaries for degumming [15,16,17,18]. However, these methods exhibited low gum removal rates and were still inadequate for producing high-grade hemp fibers. Currently, the gum structure in hemp roving is complex and remains poorly understood, leading to a limited understanding of the essential enzyme types required for bio-degumming. Consequently, there is a lack of strategies to further improve the bio-degumming efficiency of hemp roving.

A widely used method for analyzing lignocellulose structures involves slicing technology, specific dyes, and microscopy to examine the composition and morphology of the material. This technique has also been applied to characterize bast fibers [19,20,21]. Currently, several studies have analyzed the distribution of pectin, xylan, and mannan in hemp phloem. However, the linkages and side chains of these polysaccharides, as well as the morphology of the gum within the phloem, remain poorly understood [22,23]. Moreover, residual tissues are expected after the retting and processing of hemp stems into hemp roving. It is equally essential to elucidate the composition and morphology of these residual tissues.

This study analyzed the morphological features of hemp stem, roving, and refined fibers using scanning electron microscopy (SEM), identifying the tissues that needed to be removed during retting and degumming. Then, the compositions and branched-chain structures of polysaccharides in various tissues of the hemp stem, roving fibers, and refined fibers were investigated using a laser scanning confocal microscope (LSCM) combined with immunofluorescence (IF). Based on these analyses, the structure of hemp roving, along with the enzymes necessary for bio-degumming, and their synergistic effects were identified. These findings are expected to benefit the modification of degumming enzymes and microorganisms, as well as enhance the hemp retting and bio-degumming processes.

## 2. Materials and Methods

### 2.1. Materials

Hemp stems, roving, and refined fibers were supplied by Jinghua Textile Company in Xianning, Hubei Province, China. Monoclonal antibodies against plant cell wall polysaccharides and branched-chain-degrading enzymes were sourced from Megazyme (Bray, Ireland) and the Plant Probes network (https://plantcellwalls.leeds.ac.uk/plantprobes/ (Paul Knox Cell Wall Lab, University of Leeds, UK)) [24,25,26]. Additional chemicals of analytical reagent grade were procured from Thermo Fisher (Waltham, MA, USA) and Sinopharm Group (Shanghai, China).

### 2.2. Methods

#### 2.2.1. Preparation of Slices

The slices were prepared using the paraffin technique, as previously described by Behr [20]. In brief, the samples were fixed with a fixative containing 70% formalin and alcohol in a 1:10 ratio for 24 h. They were then dehydrated successively with 75% alcohol for 4 h, followed by an ethanol series consisting of 85%, 90%, and 95% for 2 h each. The samples were subsequently dehydrated with absolute alcohol twice for 30 min, followed by a 1:1 mixture of absolute alcohol and xylene for 10 min, and xylene twice for 10 min. Afterward, the samples were embedded in paraffin and subjected to cooling at −20 °C. The paraffin block was then sectioned, dried at 60 °C, and stored at room temperature. The hemp roving and refined fibers were wetted with deionized water and immediately dried at 60 °C, which caused the loose fibers to come together, forming a harder sliver, which facilitated the fixation and cutting.

#### 2.2.2. SEM Analysis

The surfaces of the hemp stem, hemp roving, and refined fibers, along with their transverse slices, were dried at 60 °C overnight, coated with gold for 100 s, and then observed through an SEM (Nova Nano450, Thermo Fisher, Waltham, MA, USA).

#### 2.2.3. Enzymatic Treatment of Slices

The enzymes involved in the enzymatic treatment are listed in Table S2. The working concentrations of pectate lyase, endo-xylanase, and endo-mannanase were set at 500 U/mL, while those for α-L-arabinofuranosidase, acetylxylan esterase, and α-galactosidase were 100 U/mL. In brief, each sample on the slice was outlined with a super PAP pen and was then treated with 0.1 mL of the corresponding enzyme solution at 37 °C for 4 h, with the enzyme solution being refreshed every hour. Subsequently, the slices were washed three times with phosphate-buffered saline (PBS, pH 7.2) to prepare for further experiments.

#### 2.2.4. Indirect Immunofluorescence Process

The indirect immunofluorescence process followed established protocols [21]. Tissue slices were blocked with 100 μL of 3% milk powder in PBS for 30 min, eluted with PBS three times, and incubated with 100 μL of antibody at 4 °C overnight; blank controls received PBS. After rinsing with PBS, the slices were incubated with fluorescein isothiocyanate (FITC, Goat anti-Rat IgG) at room temperature for 1 h, followed by incubation with 0.01% Calcofluor white for 5 min. Following PBS elution, the samples were treated with anti-fade solution and covered with a coverslip. Observation was carried out using an LSCM (FV3000, Olympus, Tokyo, Japan). Calcofluor and FITC were viewed through the 405 and FITC channels, respectively. Olympus FV31S-SW (Ver.2.3.1) software identified polysaccharides by color—blue (Calcofluor), green (pectin), yellow (xylan), and red (mannan).

## 3. Results and Discussion

### 3.1. Morphology Analysis of Hemp Stem, Roving, and Refined Fiber

**Figure 1:**
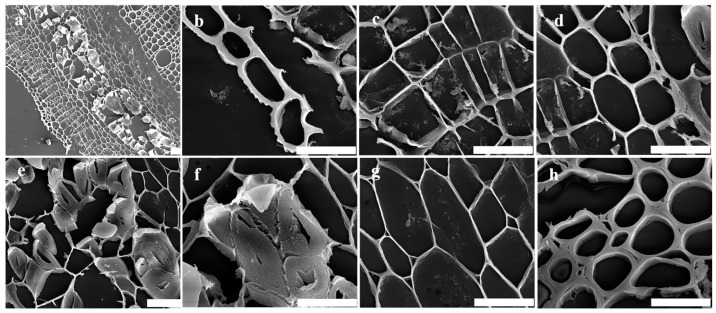
The SEM analysis of transverse sections of hemp stem; bar = 20 μm. (**a**) The transverse sections’ morphological features of hemp stem. (**b**) Epidermis. (**c**) Collenchyma. (**d**) Cortex parenchyma. (**e**,**f**) Phloem fiber. (**g**) Phloem parenchyma. (**h**) Xylem.

**Figure 2:**
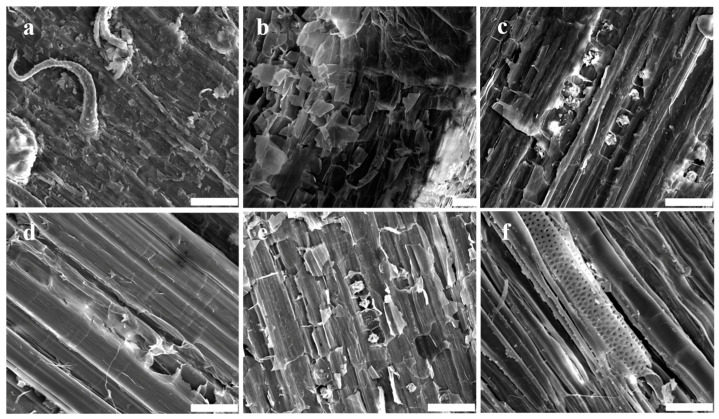
The SEM analysis of various tissues’ morphological features of hemp stem; bar = 20 μm. (**a**–**f**) Epidermis, collenchyma, cortex parenchyma, phloem fiber, phloem parenchyma, and xylem, respectively.

The morphological features of transverse sections of hemp stem are illustrated in Figure 1. The outermost to the innermost layers are depicted as follows: epidermis (e), collenchyma (cl), cortex parenchyma (cp), phloem fibers (pf), phloem parenchyma (pp), and xylem (x) (Figure 1a). The outer surface of the epidermis exhibited a thick cell wall (Figure 1b), and its surface displayed a notable trichome (Figure 2a). Both the collenchyma and cortex parenchyma showed thin cell walls and large lumens, with the notable difference that the cortex parenchyma had relatively thicker cell walls (Figure 1c,d). Moreover, the surfaces of these tissues differed significantly. The surface of collenchyma displayed fish-scale-like chunks (Figure 2b), while the cortex parenchyma exhibited a fence shape and appeared as membrane-shaped (Figure 2c). The fiber bundles were easily distinguishable due to their markedly thick cell walls and small lumen (Figure 1e,f). Each fiber bundle had a smoother surface compared to the original hemp epidermis and was encased in a chunk-shape middle lamella (Figure 2d). The phloem parenchyma had thin cell walls, but its lumen was larger than those of the collenchyma and cortex parenchyma (Figure 1g). However, its surface appeared similar to that of the cortex parenchyma, showing a fence-like shape superimposed on a membrane shape (Figure 2e). The xylem had thick cell walls with large lumen and was the thickest tissue on the slice except for the fiber bundles, making it easy to identify (Figure 1h). Additionally, its surface had a visible vascular structure (Figure 2f).

**Figure 3:**
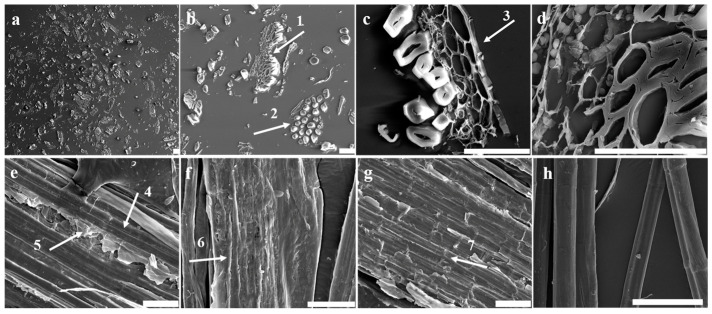
The SEM analysis of morphological features of hemp roving; bar = 50 μm. (**a**) The transverse sections’ morphological features of hemp roving. (**b**) The transverse sections of bundle fibers (arrow 2) and xylem (arrow 1). (**c**)The transverse sections of phloem parenchyma (arrow 3). (**d**) The transverse sections of xylem. (**e**–**g**) The surface morphological features of hemp roving—exposed fiber (arrow 4), chunk shape (arrow 5), stripe shape (arrow 6), fence shape (arrow 7). (**h**) The surface morphological features of refined fibers.

The transverse slice of the hemp roving exhibited a scattered distribution of tissues due to the removal of some tissue during the retting process (Figure 3a). The type of residual tissue was determined based on its shapes and connections. The scattered fiber bundle was easily distinguishable because it had very thick walls and small lumen (Figure 3b). The cell walls of the tissue connected to the fiber bundle were also thick, but the lumen was larger. Only a small amount of tissue with thinner cell walls was present between them (Figure 3b,c). Based on these results, it was concluded that the residual tissues primarily included xylem and phloem parenchyma in addition to bundle fibers; most other tissues were basically removed by the retting process. The phloem parenchyma and xylem showed fence-like and plate-like shapes, respectively. Notably, the flagella of the epidermis were not observed, further confirming the above conclusion (Figure 3e–g). Moreover, except for the fibers, no other tissues were present in the refined fibers (Figure 3h), suggesting that these remaining tissues belonged to the gums. Therefore, to determine the types of enzymes required for bio-degumming, it was necessary to identify the composition of polysaccharides in these remaining tissues and assess their changes due to retting and degumming.

### 3.2. Pectin Structure in Various Tissues of Hemp Stem Bast

**Figure 4:**
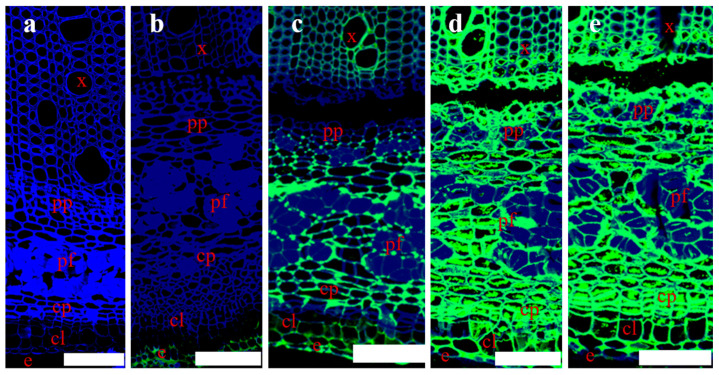
Indirect immunofluorescence analysis of various antibodies binding to pectin with different branched chains in transverse sections of hemp stem; bar = 100 μm. (**a**–**e**) Control, LM20, LM18, LM5, and LM6M. Abbreviations: e: epidermis; cl: collenchyma; cp: cortex parenchyma; pf: phloem fiber; pp: phloem parenchyma; x: xylem.

The distribution of different types of pectin in various hemp stem tissues is illustrated in Figure 4. LM18, which indicated the presence of partially esterified homogalacturonan (HG), was detected in the xylem, phloem parenchyma, cortex parenchyma, collenchyma, and epidermis, though at lower levels in the middle lamella (Figure 4c). LM20, indicative of highly esterified HG, was found exclusively in the epidermis (Figure 4b). Both LM5 and LM6M, which bind to nearly all tissues, showed weaker binding in the middle lamella (Figure 4d,e). The intense fluorescence observed in the phloem fibers of LM5 and LM6M was attributed to the presence of medullary ray in the phloem parenchyma, which facilitated nutrient transport between fiber bundles [27]. The analyses indicated that these tissues contained rhamnogalacturonan (RG) with arabinan and galactan branches, in addition to partially esterified HG.

The efficient removal of pectin was crucial for a satisfactory separation of phloem fibers from the stem during the degumming process [28,29]. The results identified the types of pectin and their branched chains within the hemp stem, revealing the presence of highly esterified HG in addition to low-esterified HG and branched RG. These pectin types were predominantly distributed in the peripheral tissues of the hemp fiber bundles, with only a minimal amount located in the middle lamella region. Previous research has also shown that HG, 1,4-galactan, and 1,5-arabinan epitopes were present at low levels in the intercellular matrices and primary walls between fibers in the phloem of hemp stem, but were virtually absent from the secondary walls of fibers [22]. This finding corroborated the above results. Therefore, it can be concluded that partially esterified HG-degrading enzymes, including endo/exo-polygalacturonases [30,31] and pectate lyases [32], as well as RG-degrading enzymes, such as endo-rhamnogalacturonases [33], endo-arabinanases [34], and endo-galactanases [35], were pivotal enzymes for hemp bio-degumming and retting. In contrast, highly esterified HG, degrading enzymes, such as the pectin methylesterases [36], may play an insignificant role. These findings contributed to clarifying which pectinase types were essential for hemp bio-degumming and retting, thus facilitating the development of efficient bio-degumming technologies.

### 3.3. Xylan and Mannan Structure in Various Tissues of Hemp Bast

**Figure 5:**
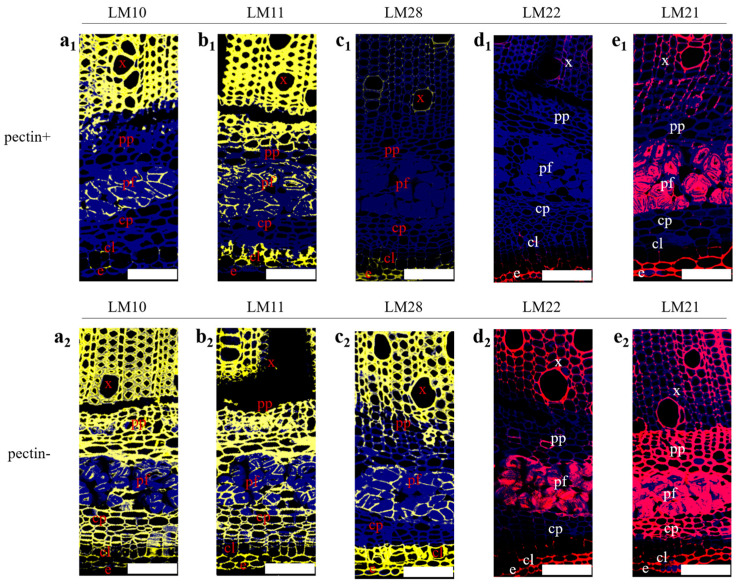
Indirect immunofluorescence analysis of various antibodies binding to xylan and mannan in transverse sections of hemp stem; bar = 100 μm. (**a_1_**–**e_1_**) Antibody distribution before pectin removal. (**a_2_**–**e_2_**) Antibodies distribution after pectin removal.

Figure 5 shows the distribution of xylan and mannan in the hemp stem bast. Before pectin removal, LM10 was bound exclusively to the xylem and middle lamella (Figure 5(a1)). LM11 displayed a broader distribution, with binding being observed in various tissues except for the cortex parenchyma (Figure 5(b1)). LM28 and LM22 exhibited binding mainly to the epidermis, although they showed weak affinity for the xylem (Figure 5(c1,d1)). LM21 was also found to bind to various tissues, with the exception of the phloem parenchyma, cortex parenchyma, and the collenchyma (Figure 5(e1)). As pectin was removed, LM10, LM11, and LM21 were able to label various tissues (Figure 5(a2,b2,e2)). LM28 demonstrated the capacity to bind to a variety of tissues, although it could not bind to the cortex parenchyma and exhibited weak binding to the phloem parenchyma (Figure 5(c2)). LM22 showed strong binding to the xylem and middle lamella, as well as weak binding to the phloem parenchyma and collenchyma, but still did not bind to the cortex parenchyma (Figure 5(d2)). The findings revealed that pectin masked xylan and mannan to varying extents across different tissues in the hemp stem bast. The masking effect of pectin on xylan and mannan in the middle lamella, cortex parenchyma, and phloem parenchyma suggested that pectin may impede the enzymatic removal of these compounds by endo-xylanase and endo-mannanase. Moreover, the distribution of the LM28 epitope indicated that xylan with glucuronic acid branching was mainly distributed in the xylem, middle lamella, and collenchyma, was less present in the phloem parenchyma, and was absent in the cortex parenchyma. The fluorescence intensity of LM21 was observed to be stronger than LM22, suggesting the presence of galactose branching in partial mannan, particularly in the phloem parenchyma and cortex parenchyma. However, since LM11 could bind to both 1,4-β-D-xylan and arabinoxylan [20], the difference in fluorescence intensity between LM11 and LM10 in hemp stem bast was not significant. Therefore, further verification of arabinose branching was required.

The above results were found to be complementary to previous studies [20,22,23,37]. The masking effect of pectin on xylan and mannan could further inhibit the hemicellulose activity, implying a synergistic relationship between pectin-degrading enzymes and hemicellulose-degrading enzymes during the retting process of the hemp stem. Additionally, hemicellulose, as the major component of gums, was composed of xylan and mannan with branched chains, which were present in the middle lamella of phloem fiber bundles and their peripheral tissues. Therefore, it could be surmised that in addition to the key enzymes responsible for the degradation of the main chains of hemicellulose, the enzymes capable of degrading the branched chains of hemicellulose also played an instrumental role in the hemp retting process.

### 3.4. Distribution of Pectin, Xylan, and Mannan in Hemp Roving and Refined Fibers

**Figure 6:**
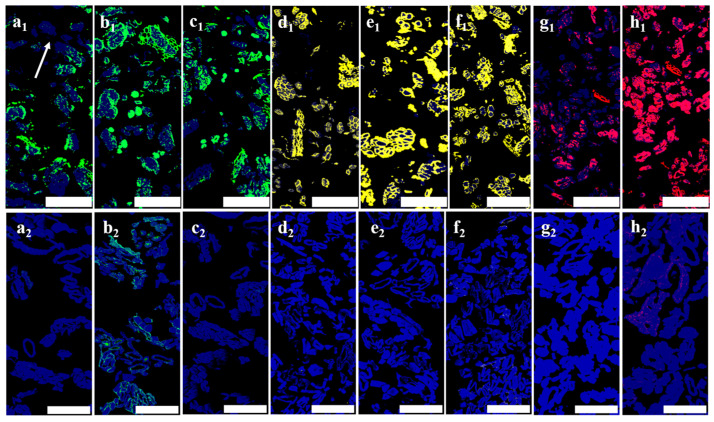
Indirect immunofluorescence analysis of various antibodies binding to xylan and mannan in transverse sections of hemp roving (**a_1_**–**h_1_**) and refined fibers (**a_2_**–**h_2_**). Bar = 100 μm. (**a**–**h**) LM18, LM5, LM6M, LM10, LM11, LM28, LM22, LM21.

Figure 6 illustrates the distribution of pectin, xylan, and mannan in the transverse sections of hemp roving. All polysaccharide epitopes were present in these sections, suggesting that pectin was only partially removed during dew retting, thereby reducing its masking effect on other polysaccharides. The distribution of the LM18, LM5, and LM6M epitopes was found to be similar in hemp roving, and was mainly present in the middle lamella, residual phloem parenchyma, and xylem (Figure 6(a1–c1)). The LM5 and LM6M epitopes were more abundant than LM18, and in some fiber bundles, LM18 fluorescence completely disappeared from the middle lamella at the arrowhead (Figure 6(a1)). LM10, LM11, and LM28 were labeled in positions similar to those observed in the hemp stem bast, including the middle lamella, residual phloem parenchyma, and xylem, with substantial accumulation (Figure 6(d1–f1)). Additionally, as previously observed in hemp stem bast, the fluorescence intensity of LM21 in hemp roving was significantly stronger than that of LM22 (Figure 6(g1,h1)). These results indicated that during the retting process, the masking effect of pectin on xylan and mannan was weakened due to the partial removal of pectin. However, hemp roving still contained a considerable amount of gum in the middle lamella, residual phloem parenchyma, and xylem. The polysaccharides in these tissues retained branched chains similar to those present before retting, suggesting that similar enzymes were required for the effective bio-degumming of hemp roving.

For an effective and controlled degumming process, the retting of hemp bast and the bio-degumming of hemp roving could be regulated, particularly in the bio-degumming step. The necessary enzymes could be introduced through the use of engineered bacteria. To circumvent the potential adverse effects of excessive enzyme introduction on the bacterium, these enzymes could be cloned into multiple strains for a combined bio-degumming process [38,39]. In addition, optimized promoter strategies could be employed to regulate enzyme expression, thereby enhancing the efficiency of hemp roving bio-degumming. Importantly, to develop a more efficient degumming enzyme system, the interactions among different enzyme types involved in the bio-degumming process of hemp roving must be further investigated.

### 3.5. Branched Chains of Xylan and Mannan and Their Influences on Degumming in Hemp Roving

**Figure 7:**
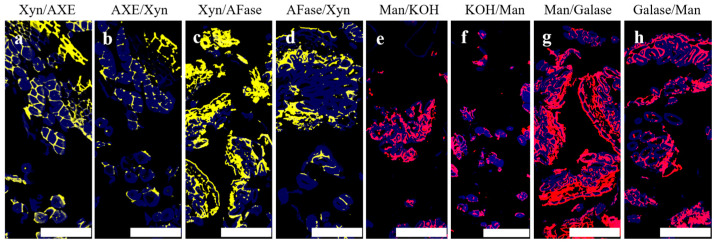
Indirect immunofluorescence analysis of LM11 (**a**–**d**) and LM21 (**e**–**h**) in transverse sections of hemp roving after different treatments; bar = 100 μm. Acetyl branching (**a**,**b**), arabinose branching (**c**,**d**) before and after removal, combined with endo-xylanase treatment. Acetyl branching (**e**,**f**), galactose banching (**g**,**h**) before and after removal, combined with endo-mannanase treatment. Abbreviations: Xyn—Endo-xylanase, AXE—Acetylxylanesterase, AFase—Arabinofuranosidase, Man—Endo-mannanase, Galase—α-Galactosidase.

The influence of polysaccharide’s branched structure on the degradation efficiency of hemicellulose in hemp roving was further analyzed. As shown in Figure 7, all branched chains were removed through the action of the corresponding branched-chain-degrading enzymes, except for the acetyl branching of mannan, which was removed using 0.1 M KOH [40]. The fluorescence intensity of LM21 and LM11 was diminished in slices where the branched chains were removed first, compared to those where they were removed later (Figure 7a–h). The results indicated that applying branched-chain-degrading enzymes prior to main-chain-degrading enzymes was more effective for the removal of xylan and mannan than the reverse order. This further confirmed that xylan in hemp roving contained not only glucuronic acid branching but also acetyl and arabinose branching, while mannan contained acetyl and galactose branching. Moreover, these branched-chain structures impeded the enzymatic hydrolysis of xylan and mannan by endo-xylanase and endo-mannanase. Therefore, to effectively remove the gums, branched-chain-degrading enzymes, in addition to hemicellulose main-chain-degrading enzymes, were required to assist in the bio-degumming of hemp roving. These included esterase, α-L-arabinofuranosidase, α-galactosidase, and α-glucuronidase.

**Figure 8:**
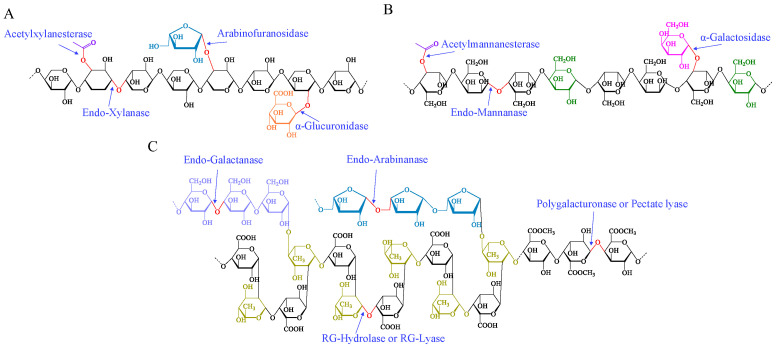
Putative structure of xylan (**A**), mannan (**B**), and pectin (**C**), as well as the enzymatic action on these polysaccharides. Abbreviations: RG—rhamnogalacturonan.

The presence of branched chains has been identified as a factor impeding the removal of hemicellulose polysaccharides during the degumming of hemp roving, indicating that this issue is prevalent in the bio-degumming of bast fibers. In bast fibers, middle lamella, phloem parenchyma, and xylem, which contained non-cellulosic polysaccharides, were identified as primary targets for degumming [41,42]. This study elucidated the polysaccharide composition, branching structures, and enzymatic action on these polysaccharides in hemp bast fibers (Figure 8). To achieve effective degumming, it was necessary to eliminate both the masking effect of pectin and the hindering effect of branched chains. The enzymes involved in the process should include highly active main-chain-degrading enzymes and a comprehensive array of branched-chain-degrading enzymes, ensuring efficient hemp bio-degumming through their synergistic interaction. Meanwhile, the composition of hemicellulose and its branched chains should be further analyzed to accurately determine the enzyme dosage required for hemp roving bio-degumming.

## 4. Conclusions

This study characterized the morphological features of hemp stem bast, hemp roving, and refined fiber; clarified the composition and branching of polysaccharides; and demonstrated their inhibitory effects on the bio-degumming of hemp roving. The enzymes required for hemp bio-degumming and their synergistic effects were identified. After retting, the remaining tissues in hemp roving included the middle lamella, phloem parenchyma, and xylem, apart from fibers, while other tissues were largely removed. The middle lamella, phloem parenchyma, and xylem, which need to be removed during degumming, displayed chunk-like, fence-like, and plate-like morphologies, respectively. The distribution of pectin, xylan, and mannan was observed in these tissues. Pectin included low-esterified homogalacturonan, as well as rhamnogalacturonan with galactan and arabinan branches. Xylan exhibited acetyl, arabinose, and glucuronic acid branches, while mannan exhibited acetyl and galactose branches. The masking effect of pectin and the presence of branched chains impeded the removal of xylan and mannan by endo-xylanase and endo-mannanase during hemp roving bio-degumming. Consequently, the requisite enzymes and their synergistic effects for hemp roving degumming were identified. Pectate lyase and rhamnogalacturonan-degrading enzymes facilitated the removal of pectin. Subsequently, xylan and mannan were removed by endo-xylanase and endo-mannanase, requiring the cooperative action of branched-chain-degrading enzymes, including esterase, α-L-arabinofuranosidase, α-galactosidase, and α-glucuronidase. These findings provide valuable insights for optimizing degumming enzymes and genetically modifying degumming strains.

## Data Availability

The data presented in this study are available within this article.

## Supplementary Materials

The following supporting information can be downloaded at: https://www.mdpi.com/article/10.3390/polym16243592/s1. Figure S1: Indirect immunofluorescence analysis of the pectin removal in transverse sections of hemp stem. Table S1: The antibodies combining various pectin, xylan, and mannan with different branched chains. Table S2: Enzymes used for slices treatment.

## Abbreviations

SEM	Scanning electron microscope
LSCM	Laser scanning confocal microscope
IF	Immunofluorescence
LM18	Antibody binding to partially esterified HG
LM20	Antibody binding to highly esterified HG
LM6M	Antibody binding to 1,5-α-L-arabinan of RG
LM5	Antibody binding to 1,4-β-D-galactan of RG
LM10	Antibody binding to 1,4-β-D-xylan
LM11	Antibody binding to arabinoxylan
LM28	Antibody binding to glucuronic acid branching of xylan
LM22	Antibody binding to 1,4-β-D-mannan
LM21	Antibody binding to galactomannan
