# Supplementary material for: Clarification of Bio-Degumming Enzymes Based on a Visual Analysis of the Hemp Roving Structure
Source: Polymers (Basel). 2024 Dec 22;16(24):3592. doi: 10.3390/polym16243592 (PMC11678562; doi:10.3390/polym16243592)
Supplement: Supplementary file 1 [file polymers-16-03592-s001.zip › polymers-3321780-supplementary.pdf]

Supplementary information

Clarification of the bio-degumming enzymes based on visual analysis of the hemp roving structure

Tianyi Yu[a,b,+], Pandeng Li[a,b,+], Tong Shu[a,b], Tingting Liu[a,b], Chunhua Fu[a,b,*], Longjiang Yu[a,b]

a. Institute of Resource Biology and Biotechnology, Department of Biotechnology, College of Life Science and Technology, Huazhong University of Science and Technology, Wuhan 430074, China

b. Key Laboratory of Molecular Biophysics, Ministry of Education, Huazhong University of Science and Technology, Wuhan 430074, China

\+ These authors contributed equally to the work.

* To whom correspondence should be addressed: *fuchunhua@mail.hust.edu.cn*

Supplementary Tables

Table S1. The antibodies combining various pectin, xylan, and mannan with different branched chains

Antibodies
LM18 (Partially- esterified HG)
LM20 (Highly- esterified HG)
LM6M (1,5-α-L-arabinan of RG)
LM5 (1,4-β-D-galactan of RG)
LM10 (1,4-β-D-xylan)
LM11 (arabinoxylan)
LM28 (glucuronic acid branching of xylan)
LM22 (1,4-β-D-mannan)
LM21 (galactomannan)

HG homogalacturonan, RG Rhamnogalacturonan.

Table S2. Enzymes used for the slices treatment

Enzymes	Company
Endo-xylanase, Xyn	Megazyme, CAS#: 9015-75-2
Endo-mannanase, Man	Megazyme, CAS#: 37288-54-3
Pectate lyase, PL	Megazyme, CAS#: 9015-75-2
Acetylxylan esterase, AXE	Megazyme, CAS#: 188959-24-2
α-L-Arabinofuranosidase, α-AFase	Megazyme, CAS#: 9067-74-7
α-Galactosidase, α-GAL	Megazyme, CAS#: 9025-35-8

Supplementary Figure

LM18

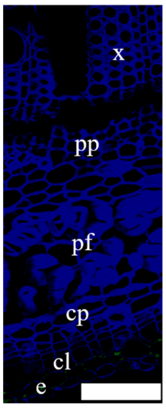

Fig.S1 Indirect immunofluorescence analysis of the pectin removal in transverse sections of hemp stem, bar=100 μm.
